# Supplementary material for: Heterogeneity in Preclinical Alzheimer’s Disease Trial Cohort Identified by Image-based Data-Driven Disease Progression Modelling
Source: medRxiv. 2023 Feb 10:2023.02.07.23285572. Preprint. [Version 1] doi: 10.1101/2023.02.07.23285572 (PMC9934776; doi:10.1101/2023.02.07.23285572)
Supplement: 1 [file NIHPP2023.02.07.23285572v1-supplement-1.pdf]

medRxiv preprint doi: https://doi.org/10.1101/2023.02.07.23285572; this version posted February 10, 2023. The copyright holder for this preprint (which was not certified by peer review) is the author/funder, who has granted medRxiv a license to display the preprint in perpetuity.
It is made available under a CC-BY 4.0 International license .

Supplementary Material

eMethods 1: Alzheimer's Disease Neuroimaging Initiative (ADNI)
Data used in the preparation of this article were obtained from the Alzheimer's Disease Neuroimaging Initiative (ADNI) database (adni.loni.usc.edu). The ADNI was launched in 2003 as a public-private partnership, led by Principal Investigator Michael W. Weiner, MD. The primary goal of ADNI has been to test whether serial magnetic resonance imaging (MRI), positron emission tomography (PET), other biological markers, and clinical and neuropsychological assessment can be combined to measure the progression of mild cognitive impairment (MCI) and early Alzheimer's disease (AD). For up-to-date information, see www.adni-info.org. MRI scans were downloaded from LONI on 27th February 2022.

eMethods 2: A4 Amyloid & Tau PET Protocols
Florbetapir PET scans were collected from 50–70 minutes post-injection and were generally reconstructed in 4x5-minute frames, which were aligned and averaged into a single 3D NIFTI-formatted image suitable for SUVr analysis. Flortaucipir PET scans were collected 90-110 minutes post-injection. The preprocessing/alignment was performed by the A4 Study investigators before data release, and so the AmyPET pipeline implemented here did not include the preprocessing of raw PET count data.

medRxiv preprint doi: https://doi.org/10.1101/2023.02.07.23285572; this version posted February 10, 2023. The copyright holder for this preprint (which was not certified by peer review) is the author/funder, who has granted medRxiv a license to display the preprint in perpetuity.
It is made available under a CC-BY 4.0 International license .

***eTable 1** – Characteristics of the ADNI subset selected using the A4 Study inclusion criteria. The subset was assigned a subtype by the A4-trained 3-subtype model. Differences across the subtypes were assessed using either an ANOVA or Pearson's χ^2 test (following Holm-Bonferroni adjustment for multiple comparisons). There was a significant difference between sex across the subtypes, but no other variables.* Abbreviations: mPACC – Modified Preclinical Alzheimer Cognitive Composite; MMSE – Mini-Mental State Examination; CDR-SB – Clinical Dementia Rating Sum of Boxes

**Characteristic**	**Overall Subset**	**Subtype Zero (sub-threshold)**	***Typical* Subtype**	***Cortical* Subtype**	***Subcortical* Subtype**	**Adjusted P-value (across subtypes)**
No. individuals	731	390	118	120	103	
Age, yrs, mean (SD)	72.9 (6.2)	73.3 (6.4)	71.7 (5.7)	71.7 (4.1)	72.0 (4.4)	.003 [a]
Female, (%)	407 (55.7)	143 (36.7)	91 (77.1)	18 (72.0)	23 (76.7)	<.001 [b]
Education, yrs, mean (SD)	16.5 (2.6)	16.8 (2.4)	15.9 (2.7)	16.7 (2.9)	16.7 (2.8)	.009 [a]
APOE ε4 alleles (%)						
0	494 (67.7)	271 (69.5)	75 (63.6)	76 (72.0)	72 (66.7)	1.00 [b]
1	198 (27.1)	102 (26.2)	37 (31.4)	32 (20.0)	27 (33.3)	
2	21 (2.9)	10 (2.6)	2 (1.7)	6 (8.0)	3 (0.0)	
Missing	18 (2.5)	7 (1.8)	4 (3.4)	6 (0.0)	1 (0.0)	
mPACC, mean (SD)	-0.13 (2.43)	-0.14 (2.35)	-0.22 (2.51)	0.22 (2.39)	-0.37 (2.63)	1.00 [a]
MMSE score, mean (SD)	29.08 (1.08)	29.13 (1.10)	29.02 (1.02)	29.07 (1.01)	29.00 (1.14)	1.00 [a]
CDR-SB, mean (SD)	0.04 (0.14)	0.04 (0.14)	0.04 (0.13)	0.04 (0.14)	0.04 (0.13)	1.00 [a]

[a] ANOVA

[b] Pearson's χ^2

medRxiv preprint doi: https://doi.org/10.1101/2023.02.07.23285572; this version posted February 10, 2023. The copyright holder for this preprint (which was not certified by peer review) is the author/funder, who has granted medRxiv a license to display the preprint in perpetuity.
It is made available under a CC-BY 4.0 International license .

eTable 2 *– Comparison of A4 Aβ+ group and the ADNI subset selected using the A4 Study inclusion criteria. Differences between the cohorts were assessed using either an ANOVA or Pearson's χ^2 test (following Holm-Bonferroni adjustment for multiple comparisons) on variables available in both cohorts relevant to this study.* Abbreviations: MMSE – Mini-Mental State Examination; CDR-SB – Clinical Dementia Rating Sum of Boxes

**Characteristic**	**A4 Aβ+ Group**	**ADNI (A4-selected) Subset**	**Adjusted P-value (across subtypes)**
No. individuals	1240	731	
Age, yrs, mean (SD)	72.0 (4.9)	72.9 (6.2)	.002 [a]
Female, (%)	730 (58.9)	407 (55.7)	.33 [b]
Education, yrs, mean (SD)	16.5 (2.8)	16.5 (2.6)	.51 [a]
APOE ε4 alleles (%)			
0	506 (40.8)	494 (67.6)	< .001 [b]
1	621 (50.1)	198 (27.1)	
2	101 (8.2)	21 (2.9)	
Missing	12 (1.0)	18 (2.5)	
MMSE score, mean (SD)	28.74 (1.28)	29.08 (1.08)	.03 [a]
CDR-SB, mean (SD)	0.06 (0.17)	0.04 (0.14)	< .001 [a]

[a] ANOVA

[b] Pearson's χ^2
